# Insulin resistance induced by *de novo* pathway–generated C16-ceramide is associated with type 2 diabetes in an obese population

**DOI:** 10.1186/s12944-022-01634-w

**Published:** 2022-02-20

**Authors:** Shahanas Chathoth, Mona H. Ismail, Hanan M. Alghamdi, Hazem Mohamed Zakaria, Khairi Ahmed Hassan, Saeed Alshomimi, Chittibabu Vatte, Cyril Cyrus, Hind S. Alsaif, Ahmed Mostafa, Heba Shaaban, Amein Al Ali

**Affiliations:** 1 Department of Biochemistry, College of Medicine, Imam Abdulrahman Bin Faisal University, PO Box 1982, 31441 Dammam, Saudi Arabia; 2 Division of Gastroenterology, College of Medicine, King Fahd Hospital of the University, Imam Abdulrahman Bin Faisal University, PO Box 40149, 31952 Al-Khobar, Saudi Arabia; 3 Department of Surgery, College of Medicine, King Fahd Hospital of the University, Imam Abdulrahman Bin Faisal University, 31952 Al-Khobar, Saudi Arabia; 4 Department of Radiology, College of Medicine, King Fahd Hospital of the University, Imam Abdulrahman Bin Faisal University, 31952 Al-Khobar, Saudi Arabia; 5 Department of Pharmaceutical Chemistry, College of Clinical Pharmacy, Imam Abdulrahman Bin Faisal University, PO Box 1982, 31441 Dammam, Saudi Arabia

**Keywords:** Obesity, Diabetes, Sphingolipids, Ceramide, Insulin resistance, Serine palmitoyl transferase, *De novo* pathway

## Abstract

**Background:**

Obesity and diabetes are two chronic metabolic diseases whose prevalence is increasing at an alarming rate globally. A close association between obesity, diabetes, and insulin resistance has been identified, and many studies have pinpointed obesity as a causal risk factor for insulin resistance. However, the mechanism underlying this association is not entirely understood. In the past decade, ceramides have gained attention due to their accumulation in certain tissues and their suggested role in initiating insulin resistance. This study aims to determine the association of specific ceramides and their major metabolizing enzymes with obesity-associated insulin resistance.

**Methods:**

The samples comprised subcutaneous adipose tissues collected from three cohorts: lean non-diabetic (controls; *n* = 20), obese-non-diabetic (*n* = 66), and obese-diabetic (*n* = 32). Ceramide levels were quantified using LC-MS/MS and mRNA expression level for different enzymes were estimated using real-time PCR-based RNA expression analysis.

**Results:**

C16-ceramide (*P* = 0.023), C16-dihydro-ceramide (*P* < 0.005), C18-dihydro-ceramide (*P* = 0.009) and C24-ceramide (*P* = 0.040) levels were significantly increased in the obese cohort compared to the control group. However, stratification of the obese group revealed a significant increase in the C16-ceramide levels (*P* = 0.027) and mRNA over expression of the serine palmitoyl transferases enzyme subunit SPT1 (*P* < 0.005) in the obese-diabetic cohort compared to the obese-non-diabetic cohort.

**Conclusions:**

The present study indicates that C16-ceramide plays a pivotal role in inducing insulin resistance. Overexpression of *SPT1* in the obese-diabetic group and its positive correlation with C16-ceramide suggest that C16-ceramide was generated through the *de novo* pathway.

**Supplementary Information:**

The online version contains supplementary material available at 10.1186/s12944-022-01634-w.

## Background

The frequency of obesity and diabetes is growing at an alarming rate worldwide. The quality of life of affected individuals is greatly reduced as a result of these two metabolic diseases. As per the World Health Organization (2016), among the 1900 million overweight adult individuals, approximately 650 million are identified as obese (BMI ≥ 30 kg/m^2^) [1]. Similarly, the prevalence of type 2 diabetes (T2D) has increased significantly, affecting 422 million people worldwide and accounting for 1.6 million deaths per year [2]. A close association has been identified between obesity, diabetes, and insulin resistance (IR). Multiple studies have pinpointed obesity as a causal risk factor for IR, a pathophysiological state in which peripheral tissues show a decreased response to insulin action [3, 4]. Insulin resistance results in raised blood glucose levels and hence precedes the onset of T2D. Various mechanisms have been reported to explain the development of IR, including dysfunction of mitochondria, overproduction of reactive oxygen species, induction of endoplasmic reticulum stress, activation of inflammatory pathways, and accumulation of bioactive lipids [5]. However, the exact mechanism(s) underlying this phenomenon is not entirely understood.

Recent studies have reported a prominent role for sphingolipid metabolites and their regulating enzymes in inducing IR [6–8]. Ceramides, which are bioactive sphingolipid metabolites, play a central role in sphingolipid metabolism and are involved in overnutrition, inflammation, and metabolic dysregulation. Ceramides are produced through either the condensation of serine with palmitoyl-CoA (the *de novo* pathway), sphingomyelin hydrolysis, or through salvage pathways [9]. In addition to their role as cell membrane constituents, ceramides also act as second messengers in many cellular signaling pathways that regulate apoptosis, proliferation, differentiation, adhesion, motility, growth arrest, and senescence [10]. Ceramides are also involved in regulating the activity of many enzymes, such as kinases and phosphatases, and modulating the activity of specific transcription factors [11, 12].

Recent studies have investigated the role of ceramides as a causal factor for obesity and obesity-associated T2D, as elevated levels of certain ceramides initiate IR [7]. These ceramides negatively influence insulin action by limiting Akt/protein kinase B, the principal regulator of anabolic metabolism and glucose acceptance [13], through a protein kinase C zeta-dependent mechanism [14, 15]. In the past two decades, determining the involvement of ceramides in the induction of obesity-associated IR has been the key aim of multiple studies [16]. In an obese rodent model, improved glucose tolerance was observed when ceramide synthesis and accumulation were inhibited [17]. Subsequent studies conducted in human cell line and rodent models demonstrated that manipulating the pathways for ceramide synthesis or deprivation using pharmacologic and genetic approaches had an intense effect on insulin sensitivity [18]. Similarly, in humans, accumulation of ceramides (specifically, C16-ceramide) in adipose tissues is associated with IR [19, 20]. In addition, studies have implicated ceramides in inducing impaired mitochondrial function, which may play a key role in IR [21].

Turpin et al. [20] reported that the ceramide-metabolizing enzyme ceramide synthase-6 (CerS6), plays a significant role in obesity-induced diabetes. Ceramide synthases (sphingosine N-acyltransferases) participate in the acylation of sphingoid bases during ceramide synthesis by the *de novo* pathway. The *de novo* synthesis pathway is initiated with the condensation of the amino acid serine with palmitoyl-CoA carried by the enzyme serine palmitoyl transferase (SPT) to produce 3-ketosphinganine. Ceramide is mainly generated through this *de novo* pathway and this initial step of *de novo* pathway is the rate-limiting step of the *de novo* pathway.

In humans, the information about pathways involved in ceramide generation and the mechanisms by which ceramides induce IR is sparse. Hence, this study evaluates the levels of specific ceramides associated with obesity-associated IR and the expression of major metabolizing enzymes to identify the specific ceramide generation pathway involved in the initiation of obesity-induced diabetes.

## Methods

### Materials and reagents

All the standards used in this study were purchased from Cayman chemicals (Ann Arbor, MI, USA) and the internal standard was procured from Avanti Polar Lipids (Alabaster, AL, USA). Methanol, acetonitrile, and isopropanol (HPLC grade) were obtained from Merck (Darmstadt, Germany). Unless stated otherwise, all chemicals were procured from Sigma-Aldrich (St. Louis, MO, USA).

### Study cohort

Subcutaneous adipose tissue samples were obtained from obese patients undergoing bariatric surgery (*n* = 98) and lean healthy controls (*n* = 20) from those undergoing cholecystectomies at King Fahd Hospital, IAU, Saudi Arabia. The obese subjects were further classified as obese-diabetic (obese-DM; *n* = 32) and obese-non-diabetic (obese-NDM; *n* = 66) based on their HbA1c and fasting blood sugar levels. The present study was approved by the institutional review board of the Imam Abdulrahman bin Faisal University (IRB # 2014-08-047). Written informed consent was obtained from all participants before sample collection. Approximately 200 mg of subcutaneous adipose tissue was collected and instantly frozen in liquid nitrogen and stored at − 80 °C until lipidomic and expression analysis was performed.

### RNA expression analysis

Approximately 100 mg of subcutaneous adipose tissue was homogenized in Trizol Reagent (Invitrogen, Thermo Fisher Scientific, Bedford, MA, USA) using a mortar and pestle. The RNeasy Mini Kit (Qiagen, Maryland, USA) was used for RNA purification followed by DNase treatment as per the manufacturer’s protocol. The RNA concentration was quantified using a NanoDrop spectrophotometer (Fisher Scientific, Bedford, MA, USA) and stored at − 80 °C until complementary DNA (cDNA) synthesis was performed. Gene specific oligo (dT) primers, Superscript III reverse transcriptase (Invitrogen, Fisher Scientific, Bedford, MA, USA), and 1 µg of RNA was used for cDNA synthesis. Gene expression was analyzed for the following enzymes: serine palmitoyl transferase 1 (*SPT1*: F-GGATTTGCCACCATAGCC, R-GAGTTACACGAGCCTTGC), acid sphingomyelinase (*SMPD1*: F-TGGTGGAGGTGTGGAGAC, R-GAAGAGGATGCGGCTGAC), neutral sphingomyelinase (*SMPD2*: F-TTGATTGATACCTTAGCCATCG, R-ATCTGACCACCTGACATAGC), acid ceramidase (*ASAH1*: F-AGCCGCTTAATGAACTGCTG, R-TGTACCATGGAACTGCACCT), neutral ceramidase (*ASAH2*: F-GGGCCTTATCAGCTGGTTTG, R-TCTCTGATACATGGCCCGTC), sphingosine kinase 1 (*SPHK1*: F- TGGCGTCATGCATCTGTTCT, R-AACCGCTGACCATCCAGAAG) and housekeeping gene *(GAPDH*: F-GAACATCATCCCTGCCTCTAC, R-GCCTGCTTCACCACCTTC) (Applied Biosystems, ThermoFisher Scientific, Massachusetts, USA).

The QuantStudio-3 Realtime PCR system (Fisher Scientific, Bedford, MA, USA) was used to assess the relative quantification levels of specific mRNAs. A final volume of 25 µL reaction mixture was prepared by mixing synthesized cDNA, TaqMan Universal PCR MasterMix (Applied Biosystems, Fisher Scientific, Bedford, MA, USA), and gene-specific primers for SYBR Green and Gene Expression probes (TaqMan, Applied Biosystems, Fisher Scientific, Bedford, MA, USA). The gene expression fold change was determined using the 2^−ΔΔct^ method. All samples were run in duplicate.

### Ceramide quantification using LC-MS/MS

*Sample preparation*: Homogenization and sample preparation for LC-MS/MS analysis was conducted as described by Bielawski et al. [22]. Briefly, 100 mg frozen adipose tissue was homogenized in homogenization buffer (50 mM Tris, 0.25 M sucrose, 0.5 mM EDTA, and 25 mM KCl, pH 7.4) using a tissue homogenizer (Polytron PT 1200 C) using a ratio of tissue weight to buffer volume of 10% (w/v). The Pierce BCA Protein Assay Kit (Fisher Scientific, Bedford, MA, USA) was used to measure the protein concentration of each sample for normalization. The 100 µL homogenate was fortified with 50 µL of internal standard solution (100 ng/mL) and the lipid extraction was performed by adding this to 2 mL of extraction mixture (water:isopropanol:ethyl acetate, 10:30:60; v:v:v), vortexing and sonicating for 30 s continuously 3 times each, and finally centrifuging at 4000 rpm for 10 min. The collected supernatant was evaporated to dryness under N_2_ gas. The dried residue was reconstituted in mobile phase A and injected onto the UHPLC system describes below.

Stock solutions of the analytes and the internal standard (C17-ceramide (d18:1/17:0)) were prepared at 1 mg/mL concentration and stored at -20 °C. A multicomponent mixture of all of the standards was prepared by diluting the stock solutions in methanol. Additional dilutions to prepare the working solutions and calibration standards were achieved by diluting the multicomponent mixture in the mobile phase’s initial composition.

*LC-MS/MS method*: A Nexera X2 UHPLC (Shimadzu, Japan) connected to a Shimadzu 8050 triple quadrupole mass spectrometer was used for LC-MS/MS analysis. LabSolutions 5.93 software was used to process the data. The chromatographic separation was performed on an Acquity UPLC® Peptide BEH C18 Column (50 mm, 2.1 mm, 1.7 μm) protected by a guard column (Acquity UPLC® BEH C18, 1.7 m VanGuard™) from Waters (Dublin, Ireland). The mobile phase consisted of 0.1% formic acid in ultrapure water (solvent A) and 0.1% formic acid in acetonitrile/isopropanol (4:3, v/v; solvent B) in gradient elution mode with a flow rate of 500 µL/min. The sample injection volume was 5 µL and the column temperature was set at 40 °C. The gradient condition for the chromatographic method is presented in supplementary table (Table S1). Under the optimized conditions, the overall run duration, including re-equilibration, was 4 min.

*MS analysis:* The electrospray ionization source operated in the positive mode was used. Quantification was achieved using multiple reaction monitoring (MRM). Flow injection analysis and the automated MRM optimization technique in LabSolutions were used to optimize MRM transitions for each analyte. MRM optimized parameters are shown in supplementary table (Table S2). Air was utilized as a heating gas, while nitrogen was used as a nebulizing and drying gas. Argon gas (Airgas, USA) was employed for dissociation in the collision cell. The parameters used to run the system and the detailed method validation procedures are given in supplementary document (see supplemental information for more details).

Upon positive identification of a target analyte, quantification of the analyte was conducted using the most intense MRM transition using matrix-matched C17-ceramide (d18:1/17:0) internal standard calibration. A total of 8 ceramide species were quantified: C16-ceramide (d18:1/16:0), C16-dihydro-ceramide (d18:0/16:0), C18-ceramide (d18:1/18:0), C18:1-ceramide (d18:1/18:1(9Z)), C18-dihydro-ceramide (d18:0/18:0), C20-ceramide (d18:1/20:0), C22-ceramide (d18:1/22:0) and C24:1-ceramide (d18:1/24:1(15Z)).

### Statistical analysis

The categorical variables are presented in tables as number and percentage, and the continuous variables are presented as the mean ± SD. *P* values < 0.05 were considered to be statistically significant. A two-sided t-test was employed to compare the average levels of biochemical parameters of the study subjects. Data were analyzed using SPSS version 20 software (IBM, USA). Prism 5 software (GraphPad, USA) was used for data analysis and graph generation.

## Results

### Baseline characteristics

The basic anthropometric characteristics, biochemical parameters, and BMIs and the mean ± SD of all parameters for all subjects are shown in Table 1. The obese subjects (*n* = 98) were between 18 and 62 years of age with a mean BMI of 45.59 ± 8.25 kg/m^2^ and the lean healthy control subjects (*n* = 20) were between 22 and 53 years of age with a mean BMI of 24.66 ± 3.24 kg/m^2^. There were significant differences in fasting blood glucose (*P* = 0.006), insulin (*P* < 0.005), and Homeostatic Model Assessment of IR (HOMA-IR) (*P* < 0.005) levels between these groups. The mean ± SD of these basic characteristics and biochemical parameters after stratifying the obese cohort into obese-NDM and obese-DM groups are presented in Table 2. Significant differences in the levels of T-bilirubin (*P* = 0.043), AST (*P* < 0.005), ALT (*P* < 0.005), GGT (*P* < 0.005) and ALP (*P* < 0.005) were observed between the obese-NDM and obese-DM groups.

**Table 1:** Baseline and demographic characteristics of lean and obese cohorts

Parameters	Lean	Obese	*P* Value
	*N* = 20	*N* = 98	
	**Mean ± SD**	**Mean ± SD**	
Age (years)	36.19 ± 9.83	35.13 ± 9.83	0.508
Sex (M : F)	1 : 1.9	1 : 2.5	
BMI (kg/m^2^)	24.66 ± 3.24	45.59 ± 8.25	< 0.005*
Waist circ. (cm)	72.64 ± 17.26	130.99 ± 12.72	< 0.005*
Hip circ. (cm)	96.05 ± 5.55	131.41 ± 10.01	< 0.005*
Cholesterol (mg/dL)	185.02 ± 73.24	187.69 ± 39.13	0.815
Triglycerides (mg/dL)	118.12 ± 80.97	117.09 ± 75.15	0.956
HDL (mg/dL)	50.35 ± 14.15	46.56 ± 13.85	0.269
LDL (mg/dL)	112.69 ± 57.20	121.92 ± 31.99	0.315
FBS (mg/dL)	94.67 ± 9.62	119.70 ± 39.30	0.006*
Insulin (mcIU/mL)	8.84 ± 2.97	17.89 ± 10.65	< 0.005*
HOMA-IR	2.09 ± 0.81	5.31 ± 3.63	< 0.005*
Albumin (g/L)	3.71 ± 0.46	3.51 ± 0.40	0.052
T-protein (g/L)	7.08 ± 0.55	7.28 ± 0.49	0.115
T-bilirubin (µmol/L)	0.55 ± 0.35	0.47 ± 0.37	0.417
LDH (IU/L)	175.80 ± 54.66	197.37 ± 50.91	0.0913
AST (IU/L)	29.35 ± 23.31	32.25 ± 31.66	0.699
ALT (IU/L)	66.60 ± 89.65	47.75 ± 44.54	0.162
ALP (IU/L)	72.35 ± 32.08	83.63 ± 27.93	0.111
GGT (IU/L)	56.35 ± 47.75	44.50 ± 32.01	0.171
PT (IU/L)	12.32 ± 0.78	12.24 ± 0.87	0.688
TSH (mIU/L)	1.46 ± 0.92	2.06 ± 1.17	0.038*

*P **< 0.05**

**Table 2:** Baseline and demographic characteristics of obese non-diabetic and obese diabetic cohorts

Parameters	Obese-NDM	Obese-DM	*P* Value
	*N* = 66	*N* = 32	
	**Mean ± SD**	**Mean ± SD**	
Age (years)	33.56 ± 9.76	39.03 ± 9.97	0.011*
Sex (M : F)	1 : 2.88	1 : 1.90	
BMI (kg/m^2^)	44.77 ± 7.56	47.30 ± 9.40	0.155
Waist circ. (cm)	128.65 ± 12.56	135.81 ± 11.84	0.008*
Hip circ. (cm)	129.55 ± 9.66	135.24 ± 9.77	0.008*
Cholesterol (mg/dL)	187.79 ± 34.10	187.49 ± 48.51	0.972
Triglycerides (mg/dL)	111.87 ± 65.55	127.85 ± 92.12	0.326
HDL (mg/dL)	48.39 ± 12.60	42.80 ± 15.68	0.060
LDL (mg/dL)	121.82 ± 29.60	122.11 ± 36.96	0.967
FBS (mg/dL)	99.65 ± 13.02	161.07 ± 43.06	< 0.005*
Insulin (mcIU/mL)	17.12 ± 10.50	19.46 ± 10.95	0.310
HOMA-IR	4.20 ± 2.63	7.62 ± 4.30	< 0.005*
Albumin (g/L)	3.49 ± 0.42	3.54 ± 0.34	0.566
T-protein (g/L)	7.25 ± 0.45	7.35 ± 0.57	0.330
T-bilirubin (µmol/L)	0.42 ± 0.24	0.58 ± 0.55	0.043*
LDH (IU/L)	194.97 ± 50.31	202.32 ± 52.57	0.506
AST (IU/L)	25.52 ± 15.07	46.45 ± 48.42	< 0.005*
ALT (IU/L)	37.98 ± 30.76	67.90 ± 60.01	< 0.005*
ALP (IU/L)	76.45 ± 20.72	98.43 ± 34.72	< 0.005*
GGT (IU/L)	36.68 ± 23.40	60.63 ± 40.73	< 0.005*
PT (IU/L)	12.21 ± 0.75	12.29 ± 1.09	0.671
TSH (IU/L)	2.11 ± 1.25	1.97 ± 1.00	0.572

*P **< 0.05**

### Ceramide levels in subcutaneous adipose tissue

**Fig. 1:**
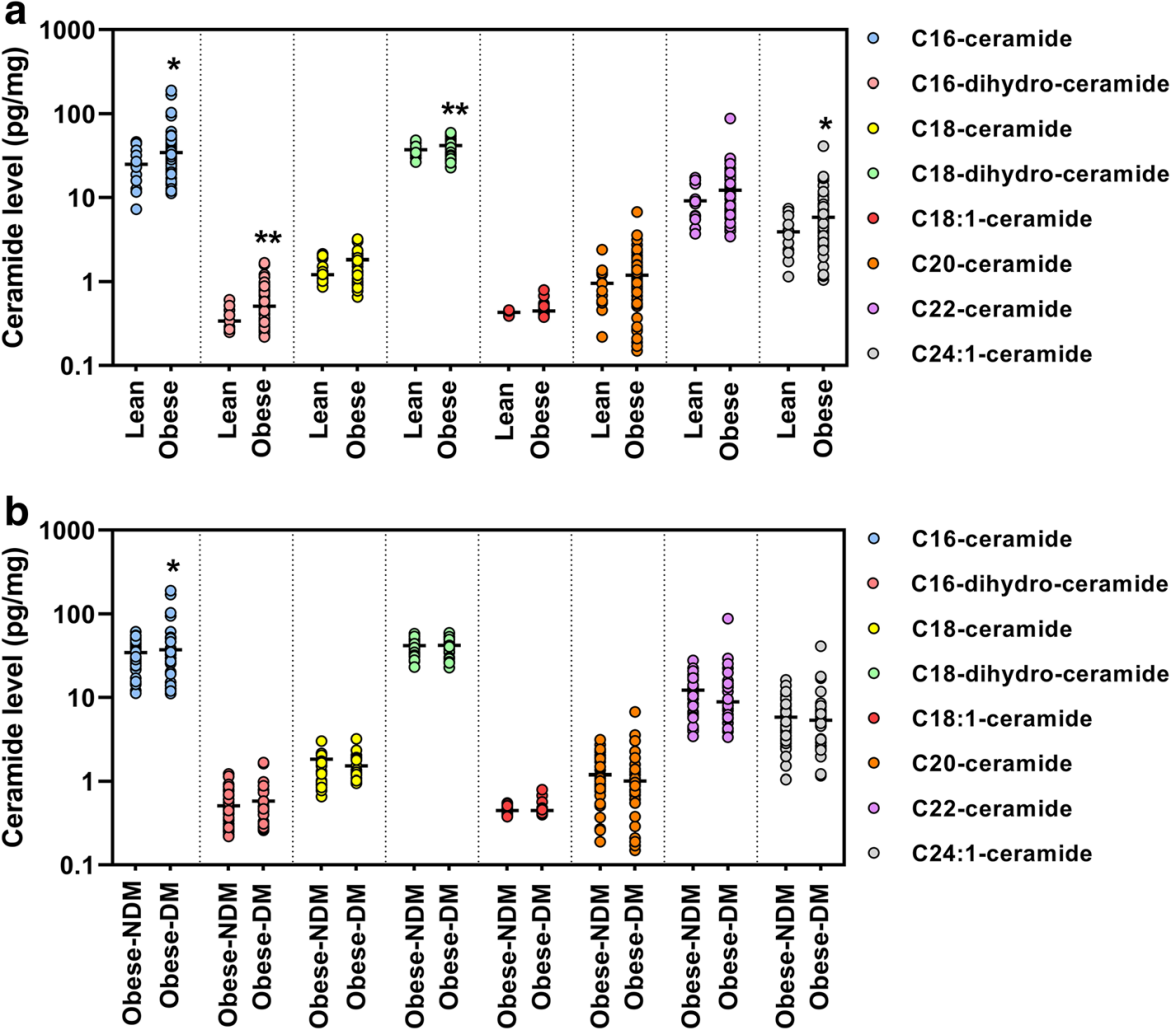
Levels of ceramides in (**A**) lean and obese groups and (**B**) obese-NDM and obese-DM groups

Levels of different species of ceramides (namely, C16-, C16-dihydro-, C18-, C18-dihydro-, C18:1-, C20-, C22-, and C24:1-ceramides) were quantified in pg/mg and tabulated (Table 3). C16-ceramide (*P* = 0.023), C16-dihydro-ceramide (*P* < 0.005), C18-dihydro-ceramide (*P* = 0.009) and C24:1-ceramide (*P* = 0.040) levels were substantially increased in the obese cohort compared to the control lean cohort. In contrast, when the obese cohort was further classified into obese-NDM and obese-DM groups, a significant difference was only observed in C16-ceramide in obese-DM group (*P* = 0.027). The levels of ceramides in lean and obese group were plotted and are shown in Fig. 1A while the comparison of ceramide levels between the obese-NDM and obese-DM groups are shown in Fig. 1B.

### mRNA expression levels of major ceramide metabolizing enzymes

**Fig. 2:**
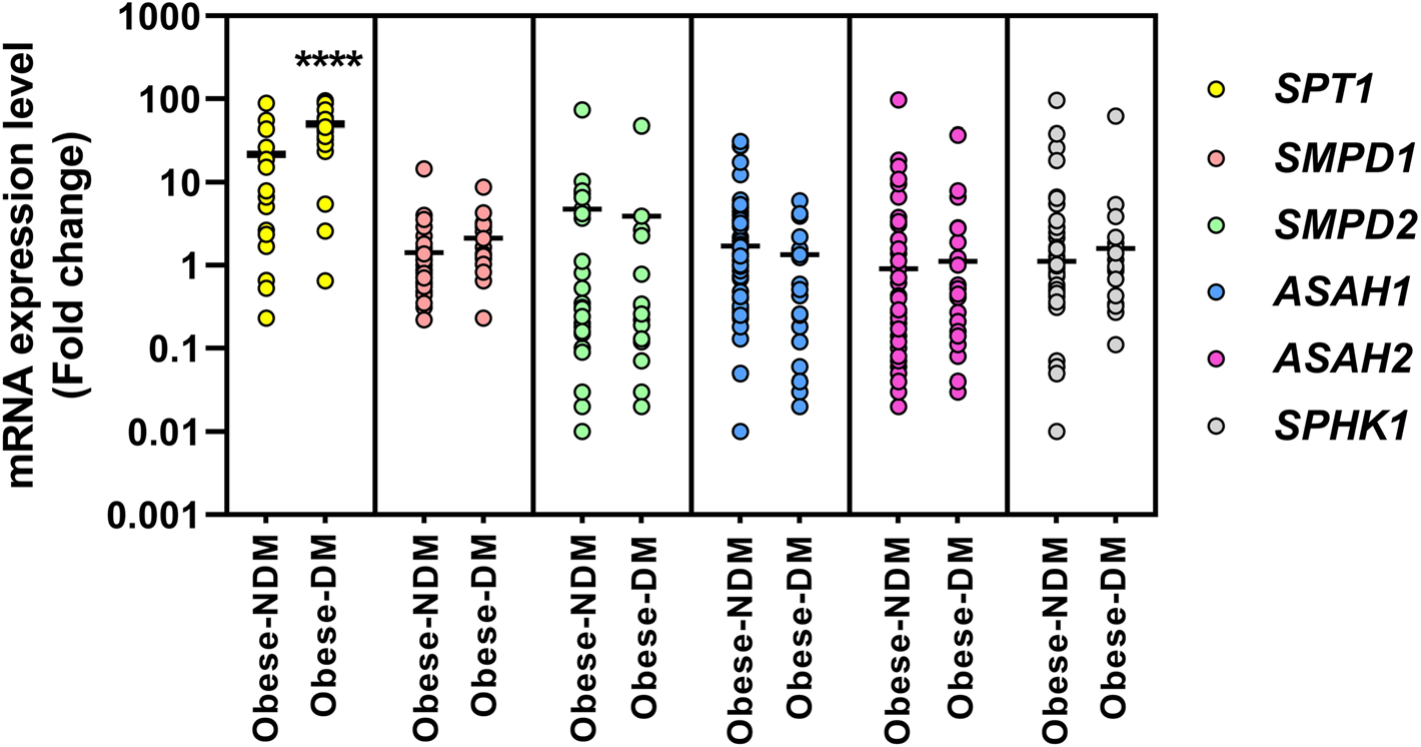
mRNA expression levels of major ceramide metabolizing enzymes in obese-NDM and obese-DM groups

The mRNA expression levels of major ceramide metabolizing enzymes, such as serine palmitoyl transferase (*SPT1*), acid- and neutral-sphingomyelinases (*SMPD1* and *SMPD2* respectively), acid- and neutral-ceramidases (*ASAH1* and *ASAH2* respectively) and sphingosine kinase 1 (*SPHK1*) was assessed for all study subjects. The fold change in mRNA expression levels was calculated for the stratified obese group (i.e., obese-NDM vs. obese-DM), and plotted as shown in Fig. 2. A significant fold change in the expression level was only observed for *SPT1*of obese-DM group (*P* < 0.005), and there was no considerable change observed in the expression levels of the other enzymes studied.

**Table 3:** Levels of ceramides quantified in lean and obese groups (A and B) and that in after stratifying obese into obese-NDM and obese-DM groups (C and D)

Ceramides	Lean (A)	Obese (B)	*P* Value (A Vs B)	Obese-NDM (C)	Obese-DM (D)	*P* Value (C Vs D)
	*N* = 20	*N* = 98		*N* = 66	*N* = 32	
	**Mean ± SD (pg/mg)**	**Mean ± SD (pg/mg)**		**Mean ± SD (pg/mg)**	**Mean ± SD (pg/mg)**	
C16-ceramide	25.09 ± 9.67	38.56 ± 25.58	0.023*	34.59 ± 12.08	46.75 ± 39.97	0.027*
C16-dihydro-ceramide	0.34 ± 0.12	0.53 ± 0.25	< 0.005*	0.51 ± 0.20	0.58 ± 0.33	0.198
C18-ceramide	1.44 ± 0.45	1.63 ± 0.48	0.114	1.63 ± 0.45	1.62 ± 0.51	0.924
C18-dihydro-ceramide	37.27 ± 4.89	41.96 ± 7.72	0.009*	41.98 ± 6.75	41.90 ± 9.34	0.961
C18:1-ceramide	0.43 ± 0.02	0.45 ± 0.07	0.153	0.45 ± 0.03	0.45 ± 0.11	0.925
C20-ceramide	0.96 ± 0.44	1.23 ± 0.86	0.089	1.20 ± 0.56	1.29 ± 1.26	0.634
C22-ceramide	9.15 ± 3.50	12.62 ± 9.39	0.109	12.30 ± 4.90	13.27 ± 14.79	0.635
C24:1-ceramide	3.92 ± 1.54	6.20 ± 4.81	0.040*	5.87 ± 2.87	6.99 ± 7.27	0.263

*P **< 0.05**

### Adipose ceramide levels and HOMA-IR

**Fig. 3:**
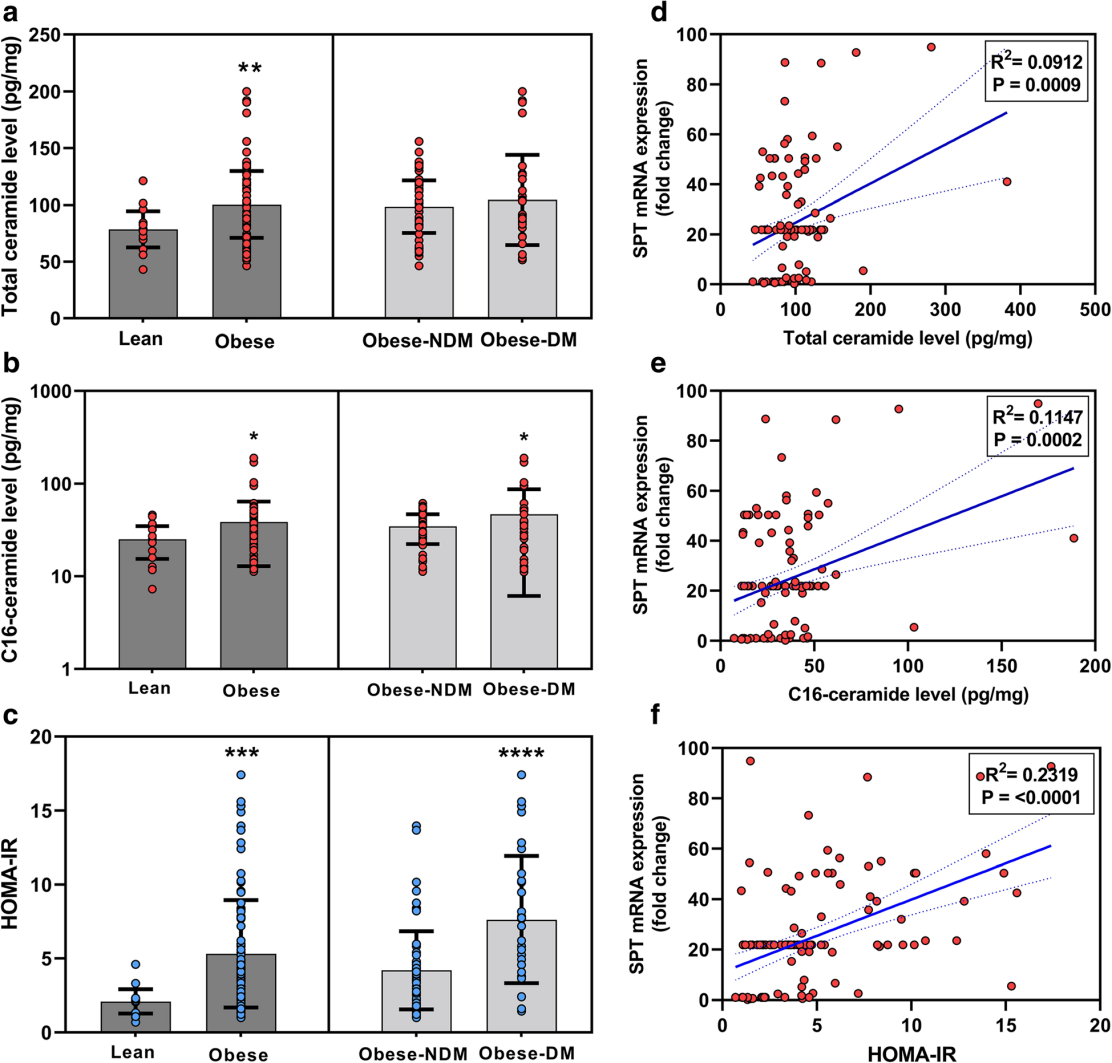
Level of Cer and HOMA-IR in lean and obese groups and obese-NDM and obese-DM groups. **A** total ceramide (**B**) C16-ceramide (**C**) HOMA-IR. Correlation analysis of SPT mRNA expression with (**D**) total ceramide levels, (**E**) C16-ceramide and (**F**) HOMA-IR

To examine whether this increased ceramide level has any association with IR, the levels of total ceramide, C16-ceramide and HOMA-IR in the lean and obese groups and the stratified obese-NDM and obese-DM groups were plotted (Fig. 3). The increased level of total ceramide in obese subjects compared to controls was significant (*P* < 0.005), but the difference in the total ceramide level was not statistically significant between obese-NDM and the obese-DM group (Fig. 3A). However, the elevated levels of C16-ceramide in the obese (compared to control) and obese-DM (compared to obese-NDM) groups was statistically significant (*P* = 0.023 and 0.027, respectively; Fig. 3B). Similarly, the increased levels of HOMA-IR in the total obese and obese-DM groups was significant compared to the control cohort and obese-NDM groups, respectively (*P* < 0.005 and *P* < 0.005, respectively), as shown in Fig. 3C. Correlation analysis was performed to assess whether *SPT1* mRNA expression was associated with ceramide levels and HOMA-IR. A significant positive correlation was observed with total ceramide, C16-ceramide, and HOMA-IR (Fig. 3D-F).

## Discussion

Ceramides, the central molecules in sphingolipid metabolism, are associated with obesity associated metabolic dysfunction. However, the link between elevated ceramide levels and obesity and obesity-induced IR remains unclear. Only a few previous studies have reported an association of elevated ceramides extracted from different sample sources, such as the circulatory system (plasma), skeletal muscle, and adipose tissue, and IR [20, 23, 24]. The importance of adipose tissue lies in its role of storing excess energy based on the systemic energy demand; and its capacity to secrete factors such as lipids, peptides, adipokines, and cytokines; and its ability to regulate metabolism in other tissues. The presence of ceramides in adipose tissue has been reported, and it was found that elevated ceramide in brown adipocytes can deregulate both insulin-regulated glucose transporter (GLUT4) expression, which plays a vital role in regulating glucose homeostasis, and glucose uptake [25–27]. Similarly, in brown adipocytes, insulin action was shown to be influenced by TNFα through *de novo* ceramide synthesis [27]. It has been observed that adipocyte hypertrophy (the excessive growth of adipocytes) in early obesity is a leading cause of IR, regardless of inflammatory responses [28].

Data from the LC-MS/MS analysis showed a significant increase in the C16-ceramide level in obese-DM compared to obese-NDM individuals. Elevation of C16-ceramide in adipose tissues has been reported in obese rodent models as well as obese human subjects, and the role of C16-ceramide in the regulation of glucose tolerance was demonstrated in knock-out mouse models of ceramide synthase 5 and 6 (CerS5 and CerS6), the essential enzymes required to synthesize C16-ceramide [20, 29]. Moreover, inhibition of ceramide synthesis using myriocin, a potent inhibitor of the enzyme SPT, or fumonisin B1, an inhibitor of ceramide synthase, or the stimulation of ceramide degradation improved insulin signaling in mice models [8, 17]. The present study found significantly elevated *SPT1* mRNA expression in obese-DM compared to obese-NDM individuals, which indicates that the activation of the *de novo* pathway for generation of C16-ceramide may be activated. A statistically significant positive correlation was noted between *SPT1* mRNA expression and total ceramide, C16-ceramide, and HOMA-IR. Notably, the stronger positive correlation between *SPT1* mRNA expression and C16-ceramide and HOMA-IR strengthens the hypothesis that C16-ceramide generated via *de novo* pathway could be inducing IR in obese individuals with T2D.

The data collected in this study clearly suggest that ceramide (C16) as a critical sphingolipid metabolite that can modify adipose tissue homeostasis function and contribute to the development of metabolic diseases. This study adds to the existing knowledge on the facilitators of obesity-related diseases, as it describes different enzymes involved in various pathways of ceramide metabolism, mainly the ceramide synthase family and SPT, which are potential drug targets via which to intervene in ceramide generation. Inhibition of these enzymes that control ceramide synthesis may also be an alternative therapeutic approach for the treatment of obesity-related diseases.

Previous studies using cell and rodent models have reported an association between elevated ceramides and IR [17, 30–32]. However, very few studies that emphasize the role of *de novo* pathway-generated ceramide in the induction of IR in humans are available, and these studies do not clearly discuss the pathways or enzymes involved [8, 33]. The role of the SPT and CerS6 enzymes in regulating ceramide levels and thereby regulating insulin sensitivity was illustrated using rodent models [8, 34], and an association between *CERS6* mRNA overexpression and elevated levels of C16-ceramide in human subjects has been reported [20]. The present study demonstrates the association between elevated *SPT1* mRNA expression and C16-ceramide levels in adipose tissues from human subjects.

To our knowledge, the present study is the first to analyze a significant number of human adipose tissue samples, as previous studies have had very low sample sizes. This strengthens the validity of the findings and increases the value of the study. The primary limitation was restricting the analysis to simple ceramides and not extending to ceramide derivatives.

## Conclusions

The present study provides further evidence that C16-ceramide plays a vital role in inducing IR. Overexpression of *SPT1* in the obese-DM group and its positive correlation with C16-cermide indicates that C16-ceramide generation in the adipose tissue of obese individual with T2D could occur through the *de novo* pathway. Hence, the study emphasizes that a targeted pharmacological approach for the downregulation of SPT could potentially reduce C16-ceramide generation and thereby combat IR among obese individuals.

## Supplementary information

**Additional file 1**

## Data Availability

The datasets used and/or analyzed during the current study are available from the corresponding author on reasonable request. The clinical data that support the findings of this study are available from the King Fahd Hospital of the University, but restrictions apply to the availability of these data, which were used under license for the current study, and so are not publicly available. Data are however available from the authors upon reasonable request and with permission of King Fahd Hospital of the University.

## Abbreviations

ALP: alkaline phosphatase
ALT: alanine aminotransferase
AST: aspartate aminotransferase
BMI: body mass index
ESI: electrospray ionization source
FBS: fasting blood sugar
FIA: flow injection analysis
GGT: gamma-glutamyl transferase
HOMA-IR: Homeostatic Model Assessment for Insulin Resistance
IR: insulin resistance
LDH: lactate dehydrogenase
MRM: multiple reaction monitoring
mRNA: messenger RNA
obese-DM: obese-diabetic
obese-NDM: obese non-diabetic
PCR: polymerase chain reaction
PKB: protein kinase B
PT: prothrombin time
SD: standard deviation
SPT: serine palmitoyl transferase
T2D: type 2 diabetes
TNF∞: tumor necrosis factor alpha
TSH: thyroid stimulating hormone
