# Supplementary material for: Insulin resistance induced by de novo pathway–generated C16-ceramide is associated with type 2 diabetes in an obese population
Source: Lipids Health Dis. 2022 Feb 20;21:24. doi: 10.1186/s12944-022-01634-w (PMC8858530; doi:10.1186/s12944-022-01634-w)
Supplement: Supplementary file 1 — Additional file 1 [file 12944_2022_1634_MOESM1_ESM.docx]

**Supplemental information**

**LC-MS/MS method for ceramide quantification**

**Table S1:** Gradient conditions for the chromatographic method.

| **Column oven temperature: 40°C, Flow rate: 500 µL min^-1^** | | | |
| --- | --- | --- | --- |
| **Mobile phase** | **A: 0.1% formic acid in ultrapure water**  **B: Acetonitrile/isopropanol (4:3, v/v) in 0.1% formic acid** | | |
| **HPLC Gradient** | Time (min) | % A | % B |
|  | 0.0 | 20 | 80 |
|  | 0.5 | 0 | 100 |
|  | 2.5 | 0 | 100 |
|  | 2.6 | 20 | 80 |
|  | 4.0 | 20 | 80 |

***The following parameters were used to run the system***

The temperatures of the ESI interface and the desolvation line were 300 °C and 250 °C, respectively. The temperature of the heat block was 400 °C. The flow rate for nitrogen nebulizing gas was 3 L min^-1^ and for drying gas and air heating gas was 10 L min^-1^. The LC retention time and the ratio of the two MRM transitions (i.e., within 20% of the ratio in the reference standards) were used to identify the target analytes. When a target analyte was positively identified, it was quantified using the highest intense MRM transition using internal standard calibration.

**Table S2:** MS/MS optimized parameters and retention times of target analytes.

|  | **Compound** | **Retention time**  **(min)** | **Polarity** | **Precursor ion** | **Product ion 1**  **(m/z)*** | **Collision Energy (eV)** | **Product ion 2**  **(m/z)** | **Collision Energy (eV)** |
| --- | --- | --- | --- | --- | --- | --- | --- | --- |
| 1 | C16-ceramide | 1.6 | + | 538.2 | 264.2 | -26 | 520.6 | -13 |
| 2 | C17-ceramide | 1.7 | + | 534.4 | 264.3 | -26 | 252.2 | -30 |
| 3 | C18-ceramide | 1.8 | + | 566.4 | 264.4 | -27 | 548.5 | -15 |
| 4 | C18:1-ceramide | 1.6 | + | 546.4 | 264.3 | -27 | 252.2 | -25 |
| 5 | C20-ceramide | 2.1 | + | 594.4 | 264.4 | -26 | 576.6 | -15 |
| 6 | C22-ceramide | 2.4 | + | 604.4 | 264.3 | -28 | 282.3 | -29 |
| 7 | C24:1-ceramide | 2.3 | + | 630.5 | 264.3 | -28 | 282.3 | -28 |
| 8 | C16-dihydro-ceramide | 1.7 | + | 540.3 | 522.5 | -20 | 284.3 | -29 |
| 9 | C18-dihydro-ceramide | 2.0 | + | 568.4 | 550.5 | -20 | 284.3 | -29 |

^*^ Precursor ion 1 used for quantification.

**Method validation**

The developed method was validated in terms of selectivity, linearity, accuracy, precision, limits of detection (LODs), limits of quantification (LOQs) and matrix effect (ME). The selectivity of the method was tested using ten adipose tissue homogenate blank samples. Two MRM transitions per analyte were recorded and their specific ratio was determined for target analyte identification. A retention time (10% around the mean value) and a ratio between the two MRM transitions with a variation of at most 20% from the average value are necessary to validate the positive detection of an analyte.

Internal standard calibration was used to estimate the linearity range. To compensate for variance during sample extraction, an internal standard (C17-ceramide) was added at a concentration of 0.75 ng mg^-1^. Each compound was analyzed in triplicate at eight different concentration levels.

Extraction recovery was estimated by spiking adipose tissue homogenate at low, medium and high concentration levels (0.5, 5 and 10 ng mg^-1^) with six replicates to verify recovery percentages. Recovery was calculated by dividing the peak areas of the target analytes after and before extraction and multiplied by 100. Intra-day precision was estimated as the RSD of six replicates (n = 6) measurements taken on the same day. Inter-day precision was assessed by running three distinct experiments over three different days and the results were represented as the RSD of these repeated measurements (n = 3 × 3).

Limits of detections (LODs) and quantifications (LOQs) were determined by analysing spiked adipose tissue homogenate and measured on the basis of signal-to-noise ratio of 3:1 and 10:1 respectively. In the case of presence of endogenous analytes in the analysed samples, LOQs were calculated as the lowest concentration that created a significant peak area when compared to the non-spiked sample extract [1].

Matrix effect (ME) was evaluated for each analyte by dividing the ratio of each analyte peak area to that of the internal standard of post extraction spiked samples by the ratio of each analyte peak area to the IS peak area prepared in solvent, then multiplied by 100. If ME (%) value is 0 %, this means there is no matrix effect. Positive values indicate ion enhancement; however, negative values indicate ion suppression.

Analytes’ stability was evaluated by using a diluted target analytes solution that was prepared in the mobile phase and stored as described in the methodology section. The solution was analyzed on different days (1, 2, 5 and 10 days) and the obtained results were then compared to those of freshly prepared standards.

Several parameters, including selectivity, linearity, accuracy, precision, LODs, LOQs, and ME were used to validate the developed method. Ten blank adipose tissue homogenate samples were employed to determine the method's selectivity. Absence of background signals above the signal to noise ratio of 3 around the retention time of the investigated analytes demonstrated the absence of false positive signals due to matrix interference, confirming the proposed method's excellent specificity.

For each individual analyte, linearity was assessed using least-squares linear regression analysis, with internal standard correction using eight points calibration, in triplicates. The calibration curves' correlation coefficients (r) for all of the investigated analytes were higher than 0.99, indicating the good linearity (Table S3).

Six replicates (n = 6) per concentration were used to calculate method accuracy and precision at three distinct concentration levels (i.e., 0.5, 5 and 10 ng/mg). In adipose tissue homogenate samples, percentage recovery varied from 97.6 to 103.2 % (Table S4). The percentage relative standard deviation (% RSD) was used to evaluate the inter-day and intra-day precision. The RSDs for the studied analytes were ≤ 4.9, which indicated satisfactory precision of the developed method (Table S4).

The absence of true real adipose tissue blank samples that is free from the target analytes is one of the key difficulties in determining experimental LOQs. This can lead to an overestimation of the LOQs for analytes present in high amounts in the blank samples. Therefore, LOQs were calculated as the lowest matrix-matched concentration that resulted in a significant increase in the chromatographic peak signal when compared to the non-spiked sample extract for those analytes found in the blank sample, as previously reported [1]. LODs and LOQs were in the range of 1.0 – 2.0 and 3.0 – 6.1 pg/mg, respectively, demonstrating the high sensitivity of the developed method.

MEs were calculated using the ratio of the responses of post extraction matrix spikes (Ratio R_post extraction)_ and the responses in pure solvent (Ratio R_pure solvent_).

*ME (%) = (Ration R_post extraction_ / Ratio R_pure solvent_) x 100*

Table 4 shows the ME for each analyte considering the range of -20% to +20% as acceptable matrix effect; however, values outside this range are considered as significant matrix effect [2]. The results revealed that no significant matrix effect was observed for all analytes.

Analytes’ stability was evaluated by calculating the percentage decrease in the analytes concentrations after being stored for 1, 2, 5 and 10 days. All analytes were found to be stable under the studied conditions.

**Table 3:** Limits of detection, limits of quantifications, linear range and linearity of individual compounds calibration curves

| Analytes | LOD (pg/mg) | LOQ (pg/mg) | Linearity range (nM) | R^2^ | Slope | Intercept |
| --- | --- | --- | --- | --- | --- | --- |
| C16-ceramide (d18:1/16:0) | 1.9 | 5.7 | 1.5 - 1000 | 0.9990 | 0.003 | -0.011 |
| C18-ceramide (d18:1/18:0) | 1.9 | 5.7 | 1.5 - 1000 | 0.9989 | 0.009 | 0.008 |
| C18:1-ceramide (d18:1/18:1(9Z)) | 1.0 | 3.1 | 0.5 - 1000 | 0.9995 | 0.029 | 0.099 |
| C20-ceramide (d18:1/20:0) | 2.0 | 6.1 | 1.0 - 1000 | 0.9989 | 0.006 | 0.041 |
| C22-ceramide (d18:1/22:0) | 1.0 | 3.0 | 0.5 - 1000 | 0.9997 | 0.022 | -0.015 |
| C24:1-ceramide (d18:1/24:1(15Z)) | 1.2 | 3.6 | 0.5 - 1000 | 0.9997 | 0.034 | -0.031 |
| C16-dihydro-ceramide (d18:0/16:0) | 1.8 | 5.6 | 1.5 - 1000 | 0.9990 | 0.009 | -0.003 |
| C18-dihydro-ceramdie (d18:0/18:0) | 1.4 | 4.4 | 0.5 - 1000 | 0.9994 | 0.017 | -0.009 |

**Table 4:** Accuracy, inter-day precision, intra-day precision and matrix effect (ME) of the developed LC-MS/MS method

| **Analytes** | **Accuracy (%)** | | |  | **ME (%)** |  | **Inter-day Precision* (%RSD)** | | |  | **Intra-day Precision* (%RSD)** | | |
| --- | --- | --- | --- | --- | --- | --- | --- | --- | --- | --- | --- | --- | --- |
|  | **Low** | **Medium** | **High** |  |  |  | **Low** | **Medium** | **High** |  | **Low** | **Medium** | **High** |
| C16-ceramide (d18:1/16:0) | 101.4 | 102.7 | 102.6 |  | -3.5 |  | 2.3 | 1.9 | 1.2 |  | 2.5 | 1.6 | 2.1 |
| C18-ceramide (d18:1/18:0) | 98.3 | 99.6 | 100.3 |  | -9.4 |  | 4.2 | 3.8 | 3.1 |  | 2.7 | 2.7 | 2.1 |
| C18:1-ceramide (d18:1/18:1(9Z)) | 98.2 | 99.2 | 103.2 |  | -10.2 |  | 3.7 | 4.2 | 1.5 |  | 2.6 | 2.7 | 3.1 |
| C20-ceramide (d18:1/20:0) | 98.2 | 100.3 | 97.8 |  | -11.7 |  | 3.7 | 3.9 | 2.6 |  | 4.2 | 3.2 | 2.9 |
| C22-ceramide (d18:1/22:0) | 100.8 | 100.2 | 98.1 |  | -4.2 |  | 3.1 | 1.5 | 1.9 |  | 2.4 | 1.2 | 0.9 |
| C24:1-ceramide (d18:1/24:1(15Z)) | 98.5 | 101.2 | 102.5 |  | 3.7 |  | 4.0 | 3.2 | 2.7 |  | 3.9 | 2.1 | 2.8 |
| C16-dihydro-ceramide (d18:0/16:0) | 97.6 | 102.3 | 99.8 |  | -14.1 |  | 3.2 | 3.0 | 3.1 |  | 4.5 | 3.7 | 2.3 |
| C18-dihydro-ceramide (d18:0/18:0) | 102.7 | 99.3 | 102.1 |  | 13.9 |  | 4.6 | 3.8 | 2.9 |  | 4.9 | 2.9 | 2.4 |

* Precision was evaluated at three concentration levels (0.5, 5 and 10 ng/mg for the low, medium and high levels, respectively).


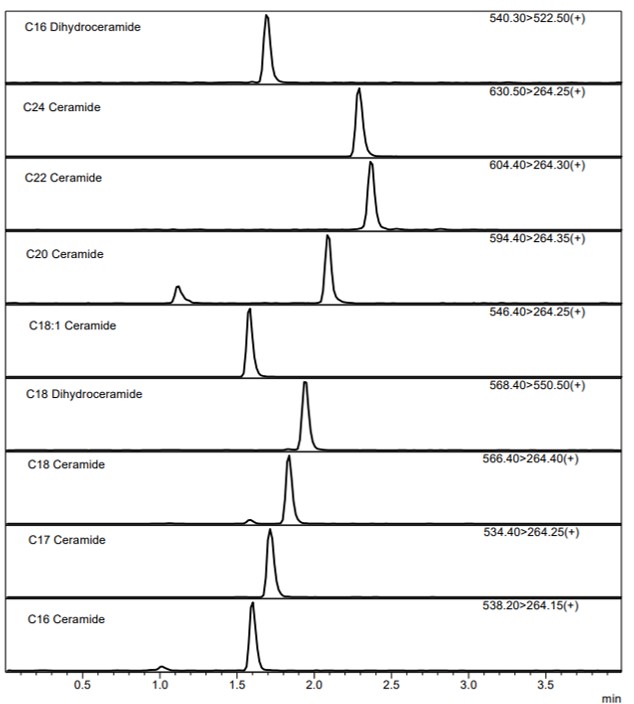


**Figure S1:** Chromatogram of ceramide standards.

1. Campos-Mañas, M.C., et al., Fast determination of pesticides and other contaminants of emerging concern in treated wastewater using direct injection coupled to highly sensitive ultra-high performance liquid chromatography-tandem mass spectrometry. Journal of Chromatography A, 2017. **1507**: p. 84-94.

2. Lopes, R.P., et al., Multiresidue determination of veterinary drugs in aquaculture fish samples by ultra high performance liquid chromatography coupled to tandem mass spectrometry. Journal of Chromatography B, 2012. **895-896**: p. 39-47.
